# Renal Vein Thrombosis in a Newborn With Abnormal Factor VIII Level: Clinical Case Report

**DOI:** 10.1097/MD.0000000000001197

**Published:** 2015-08-07

**Authors:** Agnieszka Szafranska, Agata Pajak, Katarzyna Kilis-Pstrusinska, Barbara Królak-Olejnik

**Affiliations:** From the Department of Neonatology (AS, AP, BKO); and Department of Pediatric Nephrology, Wroclaw Medical University, Wroclaw, Poland (KK).

## Abstract

Renal vein thrombosis (RVT) in neonates is a rare condition of low mortality but significant morbidity due to renal impairment.

We report the case of a male term newborn with left RVT and elevated serum factor VIII (FVIII).

The main symptoms of the patient and the important clinical findings: prompt diagnosis of RVT was possible because the classic clinical presentation of macroscopic hematuria, thrombocytopenia, and palpable flank mass were present in this newborn infant.

The main diagnoses: finally, the reason of RVT was established when the infant was 3 months of age: the increased level of FVIII was confirmed. We discuss the diagnosis, therapy, and outcome of the patient and compare with the literature.

Therapeutics interventions: however, despite anticoagulant therapy the left kidney developed areas of scarring and then atrophy.

Conclusions and outcomes: Prothrombotic defects should be considered in all patients with perinatal RVT. Elevated factor VIII as a reason of RVT in neonatal period is particularly rare. Given a poor renal outcome in children associated with elevated levels of factor VIII, consideration could be given to more aggressive antithrombotic therapy in such cases.

## INTRODUCTION

Renal vein thrombosis (RVT) is the most prevalent noncatheter-related thromboembolism in the neonatal period of life. The incidence of RVT is estimated at 2.2 per 100,000 lives or 0.5 per 1000 neonatal admissions to neonatal intensive care units (NICU). Newborn infants are predisposed to having thrombotic complications because of physiological properties of neonatal hemostasis due to decreased levels of natural anticoagulants such as antithrombin, protein C, protein S, and low levels of other components. Moreover, the susceptibility of neonatal kidney is the effect of low renal perfusion pressure and double intracapillary network in the kidney. Risk factors for the development of RVT include the following: maternal diabetes mellitus, history of perinatal asphyxia, prematurity, infection, polycythemia, and cyanotic congenital heart disease.^1^

Inherited prothrombotic abnormalities haven been described in cases reported of RVT.^2^ We report the case of a male newborn with elevated serum factor VIII (FVIII) and RVT.

## CASE REPORT

A 1-day-old male infant was transferred to the neonatology department because of RVT suspicion. He was born at 39 gestation by a primigravida via emergent cesarean section for cross-birth and uterine fibroids, with 2930 g. Apgar scores were 9/9 points at 1 and 5 minutes. Prophylactic vitamin K was administered intramuscularly. The examination of the newborn infant after birth revealed a palpable left flank mass and gross hematuria elevated C-reactive protein (CRP) (the highest value 166.71 mg/dL) and procalcitonin (PCT) (8.40 ng/mL). Intravenous antibiotic therapy was started. An abdominal ultrasound Doppler flow study revealed sonographic features of renal venous thrombosis in the left kidney: reversed end-diastolic flow in the main renal artery and significantly reduced flow in the renal vein.

Factors predisposing this neonate to RVT include maternal diabetes, early onset infection, and complicated labor. The neonate was with no family history of thrombotic disorders or fetal losses.

**FIGURE 1:**
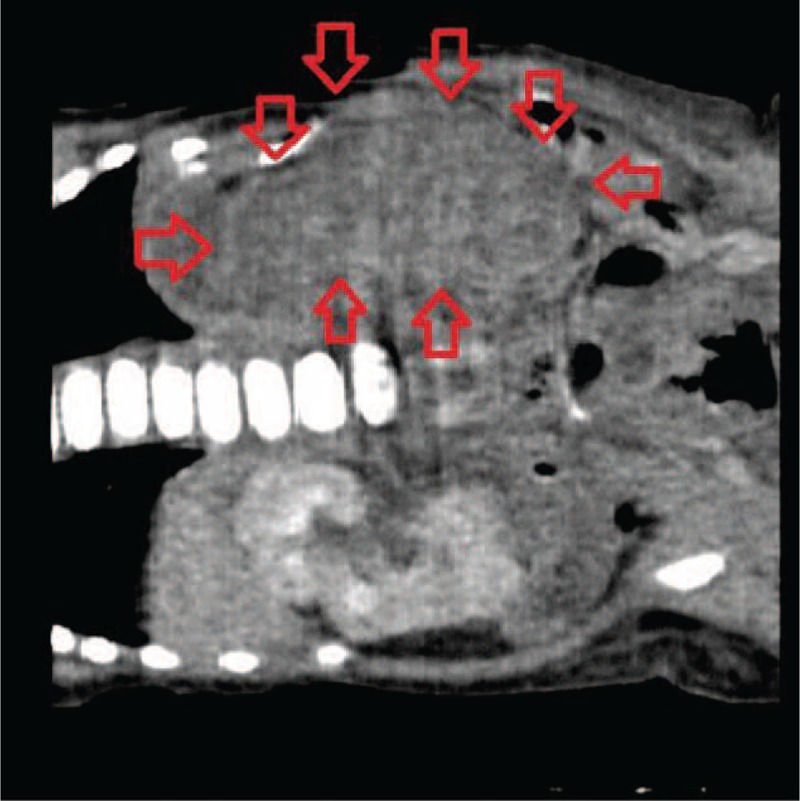
Upper abdominal CT scan after intravenous contrast medium administration. An enlarged left kidney with lack of contrast enhancement was observed.

**FIGURE 2:**
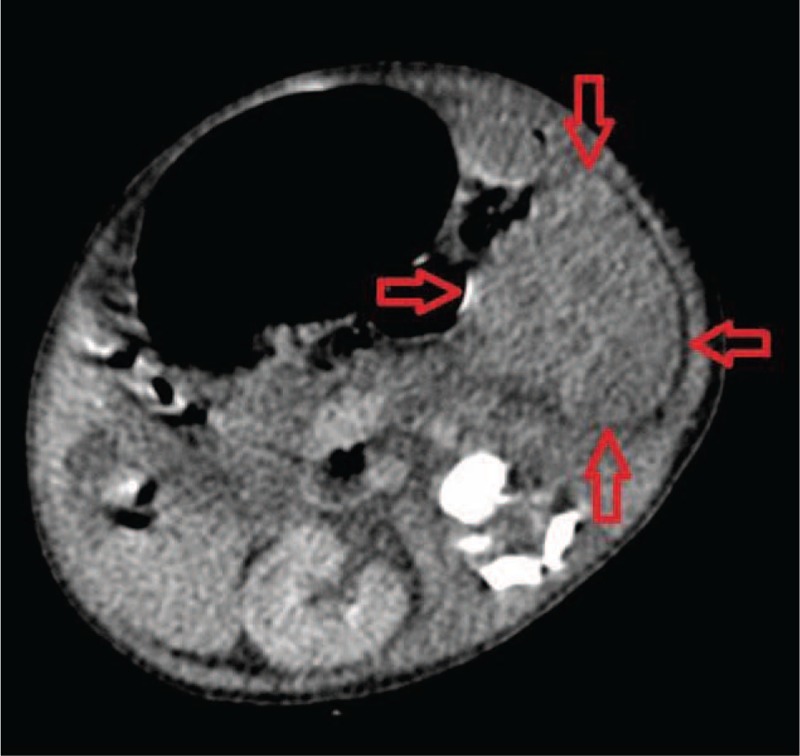
Upper abdominal CT scan after intravenous contrast medium administration. An enlarged left kidney with lack of contrast enhancement was observed.

We observed clinical deterioration during the first days of the infant's life: delayed capillary refill, mottling, paleness, hypotension, abdominal distension, feeding intolerance, and heart murmur. Abdominal ultrasound showed an enlarged left kidney (length 6.4 cm) and evidence of swelling as well as the loss of cortico-medullary differentiation (Figures 1 and 2). In differential diagnosis RVT infiltrative kidney tumor was taken into consideration. The computer tomography scan (CT-scan) of the abdomen was performed revealing left adrenal hemorrhage and massive left renal swelling (kidney size 6.3 cm × 3.3 cm). Cranial ultrasound revealed left-sided IVH II° (intraventricular hemorrhages). Initial treatment included a broad spectrum of antibiotics and anticoagulant therapy, with careful attention to fluid balance and nutrition.^3^ Initial dose was 30 U/kg/h of intravenous unfractionated heparin (UFH) and then subsequent doses 30 U/kg/h aiming for an activated partial prothrombin time ratio of 1.5–2.5. However, despite the increase in dose to 45 U/kg/h, the desired prolongation of clotting time was not achieved (International Normalized Ratio, INR 0.79–0.9). After kidney perfusion had been reestablished, the patient received 100 U/kg subcutaneous low molecular mass heparin (LMWH) twice a day. Laboratory tests of kidney function showed a transient rise in serum creatinine and urea (maximum value to 1.59 and 60 mg/dL, respectively), proteinuria, and hematuria. Inflammatory parameters gradually normalized; however, some of them never reached normal level till discharged, suggesting an ongoing inflammation. Protein C (63%), antithrombin III (50.13%) activity, and homocysteine concentration were normal for the age. The blood culture was negative.

Since therapy monitoring required frequent blood draws, red cells transfusion was recommended. During hospitalization the boy was consulted by a pediatric nephrologist and hematologist. The patient was discharged from hospital in good overall condition, on the 22nd day of life, with the recommendation for the continuation of subcutaneous heparin supply and multiprofessional care. So far, testing for heritable thrombophilic defects in the Department of Pediatric Hematology showed elevated plasma levels of factor VIII (150% of the normal level) in the third month of age.

The boy remains under the control of nephrology. Ultrasound at 3 months of life showed the left kidney was (4 cm × 2 cm) smaller than the right (5.7 cm × 2.4 cm) with loss of cortico-medullary differentiation. Renal scintigraphy revealed trace secretion by the left kidney (5%). Blood pressure was normal, serum creatinine and urea were within the normal range. After a 6-month follow-up, serum parameters of renal function and blood pressure maintained within the normal range. Ultrasound showed the right kidney 6.5 cm × 2.7 cm and the lack of left kidney (Figures 1 and 2). IVH II was every 2 weeks ultrasound controlled and the regression of changes has been observed.

## DISCUSSION

Prompt diagnosis of the patient was possible because the classic clinical presentation of macroscopic hematuria, thrombocytopenia, and palpable flank mass were present in this newborn infant. The classic triad of symptoms combined with early manifestation suggests the RVT beginning in prenatal or perinatal period.^1,4,5^ Our patient was a male infant born on term. Males are more commonly affected than females, representing about 67% of cases. It was hypothesized that males have a greater risk of congenital renal malformations, which may predispose them to a higher risk of neonatal renal vein thrombosis.^6^ The majority of neonatal RVT is unilateral with a left-sided predominance (63.6%). It was also the case in our patient.

Susceptibility of the neonatal kidney to develop thrombosis may be secondary to its low renal perfusion.^7^ When newborn infants have a central venous catheter inserted, up to 80% of the affected neonates with RVT had coexisting risk factors. Approximately 30% of these neonates were born prematurely and with signs of perinatal asphyxia or with signs of infection. Congenital heart disease had also been reported in some patients.^1,2^ Our patient's mother had prenatally gestational diabetes maternal type 1 (GDM1), but polycythemia as a risk of thrombosis was not observed.

Finally, the reason of RVT was confirmed after 3 months: the increased level of FVIII. Only 1 report had demonstrated abnormal serum FVIII level in 2 cases with RVT in that period of life.^8^ It is known that 25% of patients with venous thrombosis have FVIII levels greater than 150 U/dL. To minimalize the confounding influence of any postthrombotic acute phase response FVIII levels should be measured 3–6 months following the acute venous thromboembolism.^9^ Current data suggest that the high FVIII levels do not simply reflect a postthrombotic acute phase response, which can contribute to the elevation of fibrinogen, CRP, and FVIII. However, plasma CRP and fibrinogen levels had both corrected to normal range by 3 months in the majority of patients and FVIII levels remained persistently elevated in 72% subjects for at least 6 months.^6^ The elevated level of FVIII may be familiar and result from endothelial hyperactivity in response to vascular damage. This mechanism is not based on acute phase response.^9^ Additionally, plasma levels of FVII-VWF (complex factor VIII with factor von Willenbranda) complex are markedly influenced by ABO blood groups. Normal individuals with blood group 0 have plasma FVIII levels 25–30% lower as compared with non-0 individuals. Among the non-0 groups, AB individuals had the highest levels, followed by group B and then group A.^3^ Our patient has blood group B. So he should be in the group of medium risk of elevated levels of FVIII.

Poor prognosis in RVT results from the damage to the organ affected by thrombosis, which unfortunately was observed in our case. Accurate and early diagnosis could be an important tool for guiding clinicians in helping treatment decisions that are still controversial with different regimes used, including thrombolysis and heparin therapy as well as supportive therapy. Sonography might help in the management of RVT in neonates and also predict renal outcome.^10^ Winyard et al^10^ documented that larger perinatal kidneys had reduced long-term function. The kidneys larger than 6 cm at presentation never had a normal outcome. The first ultrasound in our boy showed the length of the affected kidney at 6.4 cm. Harris et al^6^ demonstrated that if the initial perfusion is poor, the kidney is not likely to recover completely. The presented case confirmed that the left kidney developed areas of scarring and then atrophy. Elevated factor VIII can be recognized as the next predictor of poor outcome, especially in adults.^8^

Our report has some limitations mostly related to

1. The radiologists highly suspected a renal tumor in our patient, so abdominal CT was performed, which radically lengthened the process of diagnosing RVT.
2. Although there was no consensus on the management of neonatal RVT, a multidisciplinary team has been involved.
3. As the therapeutic level of INR was not achieved, higher doses of LMWH could have been administered. Heparin therapy with unfractionated or with LMWH has been proposed for neonates with RVT. However, the renal outcomes seem not to be importantly influenced by anticoagulant treatment, as 62.5% of the affected kidneys became atrophic in neonates who were managed with LMWH.^11^ RVT in neonates often leads to irreversible damage.
4. During the unfractionated heparin therapy the anti-factor Xa levels could have been monitored. Therapeutic level is 0.35–0.7 U/mL. Anti-factor Xa should be measured 4–6 hours after administering the first dose. If within the therapeutic range, it should be checked weekly. If out of the therapeutic range, the dose should be readjusted and another check should be performed after 4–6 hours.^3^ The anti-Xa assay has been proposed by some as a better assay for heparin monitoring. Consequently, a number of medical centers have switched to the anti-Xa assay for unfractionated heparin therapy monitoring. Although anti-Xa testing offers the advantages of not being affected by coagulation factor deficiencies other than antithrombin, limited published studies document the effectiveness of anti-Xa assays for monitoring heparyn therapy. Moreover, there is some uncertainty about what constitutes the ideal approach to measuring anti-Xa levels.^12^
5. The ultrasound of abdomen with the measurement of the flow in the vessels of the kidney should be made in every patient with risk factors and thrombophilia screen performed. Thus, pregnant women with risk factors should be closely monitored and in case of any doubts prenatally, they should be directed to and remain under the care of clinic until delivery. Risk factors are sepsis, maternal diabetes, polycythaemia, dehydration, and prothrombotic mutations. Early postnatal diagnosis can prevent neonates at risk from developing the kidney disease in the future.

Very few published studies have evaluated risk factors for recurrent thrombosis in children.^8,9^ Elevated factor VIII levels are familiar or result from endothelial hyperactivity in response to vascular damage. Given a poor outcome in children associated with elevated levels of factor VIII, D-dimer, or both (>150 IU/dL and 500 ng/mL, respectively) at the time of diagnosis of thrombosis, consideration could be given to more aggressive antithrombotic therapy in such cases. Many authors have underlined that anticoagulant therapy might not have an impact on gaining the lost renal function; however, early supportive management could be beneficial in preventing severe kidney damage.
